# Normalization of glycosaminoglycan-derived disaccharides detected by tandem mass spectrometry assay for the diagnosis of mucopolysaccharidosis

**DOI:** 10.1038/s41598-019-46829-x

**Published:** 2019-07-24

**Authors:** Hsiang-Yu Lin, Yun-Ting Lo, Tuan-Jen Wang, Sung-Fa Huang, Ru-Yi Tu, Tzu-Lin Chen, Shuan-Pei Lin, Chih-Kuang Chuang

**Affiliations:** 1 Division of Genetics and Metabolism, Department of Medical Research, Mackay Memorial Hospital, Taipei, Taiwan; 2 Department of Pediatrics, Mackay Memorial Hospital, Taipei, Taiwan; 3 Mackay Junior College of Medicine, Nursing and Management, Taipei, Taiwan; 4 Department of Medicine, Mackay Medical College, New Taipei City, Taiwan; 5 Department of Medical Research, China Medical University Hospital, China Medical University, Taichung, Taiwan; 6 Department of Infant and Child Care, National Taipei University of Nursing and Health Sciences, Taipei, Taiwan; 7 Department of Laboratory Medicine, Mackay Memorial Hospital, Taipei, Taiwan; 8 College of Medicine, Fu-Jen Catholic University, Taipei, Taiwan

**Keywords:** Biochemical assays, Polysaccharides

## Abstract

Mucopolysaccharidosis (MPS) is caused by the deficiency of a specific hydrolytic enzyme that catalyzes the step-wise degradation of glycosaminoglycans (GAGs). In this study, we propose an empirical method to calculate levels of GAG-derived disaccharides based on the quantity (peak areas) of chondroitin sulfate (CS) with the aim of making a diagnosis of MPS more accurate and reducing the occurrence of false positive and false negative results. In this study, levels of urinary GAG-derived disaccharides were measured in 67 patients with different types of MPS and 165 controls without MPS using a tandem mass spectrometry assay. Two different methods of reporting GAG-derived disaccharides were assessed; normalization to urinary CS (in μg/mL), and normalization to μg/mg creatinine. CS-normalization yielded more consistent values than creatinine-normalization. In particular, levels of urinary dermatan sulfate (DS), heparan sulfate (HS), and keratan sulfate (KS) significantly varied because of changes in urine creatinine levels, which were proportional to age but inversely proportional to DS, HS, and KS measurements. Using CS-normalization revealed the actual status of DS, HS, and KS without the influence of factors such as age, urine creatinine, and other physiological conditions. It could discriminate between the patients with MPS and controls without MPS, and also to evaluate changes in GAG levels pre- and post-enzyme replacement therapy.

## Introduction

Mucopolysaccharidoses (MPSs) are a group of lysosomal storage disorders (LSDs) caused by deficiency in one specific enzyme that catalyzes the stepwise degradation of glycosaminoglycans (GAGs). Depending on the type of MPS, this deficiency leads to excessive lysosomal storage of either chondroitin sulfate (CS), dermatan sulfate (DS), heparin sulfate (HS), or keratan sulfate (KS) and results in devastating manifestations such as coarse facial features, developmental delay and decline, gibbus, hepatosplenomegaly, cardiac valve disease, umbilical and inguinal hernias, joint deformity with a restricted range of motion, airway dysfunction with complications, sleep apnea, recurrent otitis media, and premature death^1–5^. The clinical manifestations of MPS are chronic and progressive, and the initial onset of clinical signs and symptoms of MPS emerge between the ages of 18 months and 4 years, depending on disease severity^6–8^. Enzyme replacement therapy (ERT) is widely used and available for MPS I, MPS II, MPS IVA and MPS VI^5,9–14^, and trials are currently ongoing for MPS IIIB. Achieving optimal benefits from ERT requires commencing treatment before the onset of irreversible clinical presentations^5,15,16^.

Conventionally, a confirmation of MPS is achieved using a sequence of assessment profiles, including one or more typical signs or symptoms of MPS, positive first-line urinary biochemistry examinations, deficiency of leukocyte enzyme activity, and definite pathogenic variations in MPS gene alleles. Tandem mass spectrometry (liquid chromatography/tandem mass spectrometry; LC-MS/MS) is widely used to quantitatively measure levels of urinary GAG-derived disaccharides to diagnose MPS^17–19^.

The principles of using tandem mass spectrometry to measure GAG-derived disaccharides including CS, DS, HS, and KS have been described previously^17–21^. Methanolysis is used to quantify CS, DS, and HS, and keratanase II digestion is mainly used to determine urinary KS. Tandem mass spectrometry is a feasible methodology with very good false positive and false negative correlations in relation to true positives and true negatives, which can compensate for inadequate interpretations using two-dimensional electrophoresis (2-D EP) to separate MPS molecules. The LC-MS/MS assay can provide very valuable and precise information with regards to changes in the quantities of individual GAG-derived disaccharides to assist in making an initial diagnosis of MPS and also in monitoring outcomes to assess the pharmacodynamic (PD) effect on GAG levels by ERT. Since the LC-MS/MS assay is highly sensitive and specific, it can more accurately reveal the PD effect of ERT on GAG levels as compared to dye-based methods (such as dimethylmethylene blue (DMB)/creatinine (Cre) ratio)^22,23^.

The MS/MS-based method used to quantitatively measure GAG-derived disaccharides is powerful and sensitive enough to screen for highly suspected cases of MPS. However, the normalization of units is extremely important due to the high possibility of misinterpreting results if values are expressed using μg/mg creatinine. In particular, variations in urine creatinine level can result in significant variations in the levels of GAG-derived disaccharides if they are expressed in μg/mg creatinine. In addition, urine creatinine itself is a variable factor which is proportional to age but inversely proportional to levels of GAG-derived disaccharides. Moreover, urine creatinine level can be seriously affected by diet, dehydration, renal function, age, activity, body size and muscle mass^24^. Nevertheless, most MPS reference laboratories express levels of GAG-derived disaccharides in μg/mg creatinine, even though this can increase the difficulty and uncertainty in making a diagnosis, and also when evaluating the efficacy of ERT. Therefore, the rationale of this research was to propose normalizing the units of the urinary levels of three GAG-derived disaccharides (DS, HS and KS) with respect to CS (area of the CS peak) as µg/mL instead of μg/mg creatinine, with the aim of making a diagnosis of MPS more accurate and reducing the occurrence of false positive and false negative results.

## Results

As previously reported, the validation of the LC-MS/MS assay for GAG-derived disaccharides was excellent. The intra-assay and inter-assay precisions (coefficients of variance %) were both less than 10% (7.38% and 8.67%, respectively). The validation of the LC-MS/MS assay for the quantitative analysis of GAG-derived disaccharides confirmed that the method met the requirements for both MPS first-line biochemistry examinations and a confirmative diagnosis of MPS.

### The reference cut-off values of the non-MPS controls

**Figure 1:**
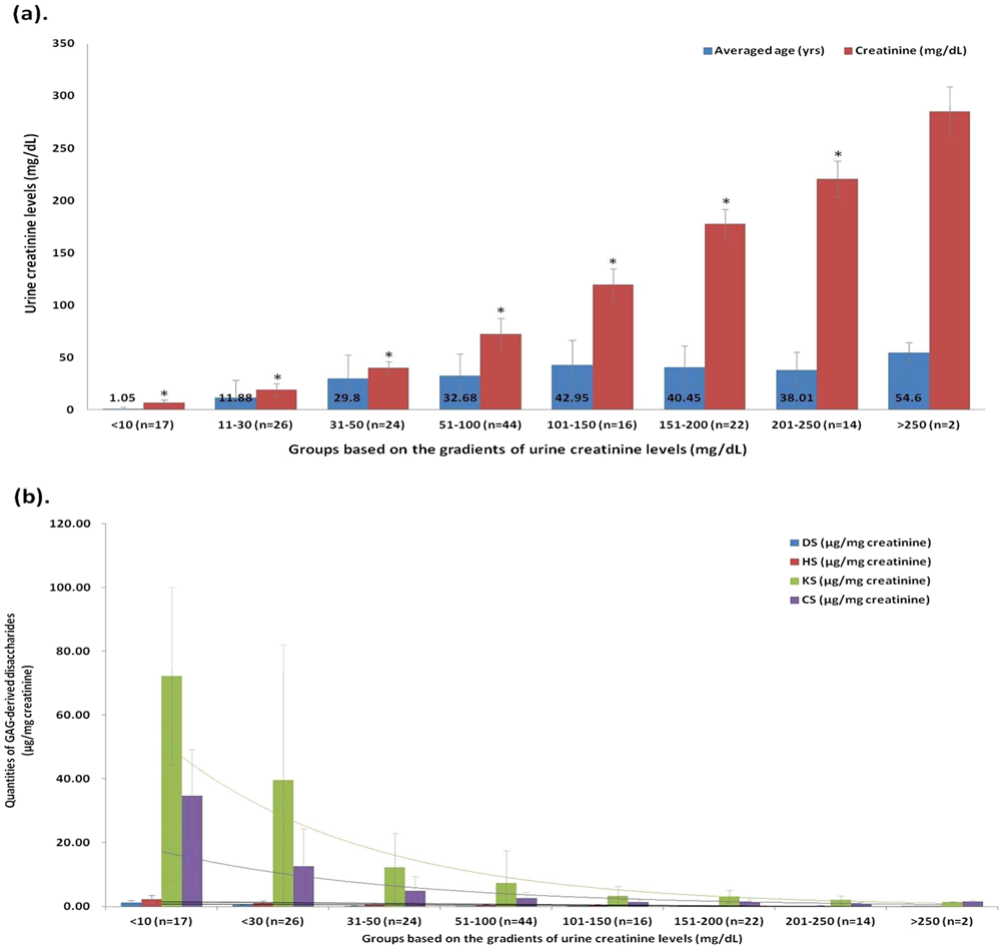
Groups of non-MPS controls were classified according to the gradients of urine creatinine levels. Urine creatinine levels were directly proportional to age (**a**); but were inversely proportional to the quantities of GAG-derived disaccharides (i.e. DS, HS, and KS) calculated using the creatinine-normalized method (**b**). The asterisk on the bar graph represents the *p*-value (<0.01) calculated using the Student’s two-sample t-test assuming unequal variance.

The mean (±SD) levels of DS, HS, and KS calculated using CS as the basis in the non-MPS controls (n = 165) were 0.10 (±0.13), 0.14 (±0.14), and 0.82 (±2.33) μg/mL, respectively, and the reference cut-off values were <0.49, <0.56, and <7.81 μg/mL, respectively, as determined in a range of +3 SD. In comparison, using urine creatinine (mg) as the basis for calculations, the levels of GAG-derived disaccharides in the non-MPS controls were larger and showed notable discrepancies due to large variations in urine creatinine levels (mean: 86.42 ± 70.84 mg/dL; range from 2.10 to 308.69 mg/dL). The mean (±SD) values of DS, HS, and KS in the non-MPS controls were 0.30 (±0.42), 0.67 (±0.81), and 4.52 (±3.09) μg/mg creatinine, respectively, and the cut-off values (+3 SD) of DS, HS, and KS were <1.56, <3.1, and <13.79 μg/mg creatinine, respectively. Urine creatinine levels were directly proportional to age, but inversely proportional to the quantities of GAG-derived disaccharides when creatinine-normalization was used for the calculations (Fig. 1a,1b). As illustrated in Fig. 1b, the lower the urine creatinine level, the higher the quantities of the GAG-derived disaccharides, particularly CS and KS. For CS, the level varied from 34.72 (<10 mg/dL creatinine) to 1.52 μg/mg creatinine (>250 mg/dL creatinine), and for KS the level varied from 72.21 (<10 mg/dL creatinine) to 1.43 μg/mg creatinine (>250 mg/dL creatinine).

### LC-MS/MS quantitative analysis of GAG-derived disaccharides in different types of MPS

**Figure 2:**
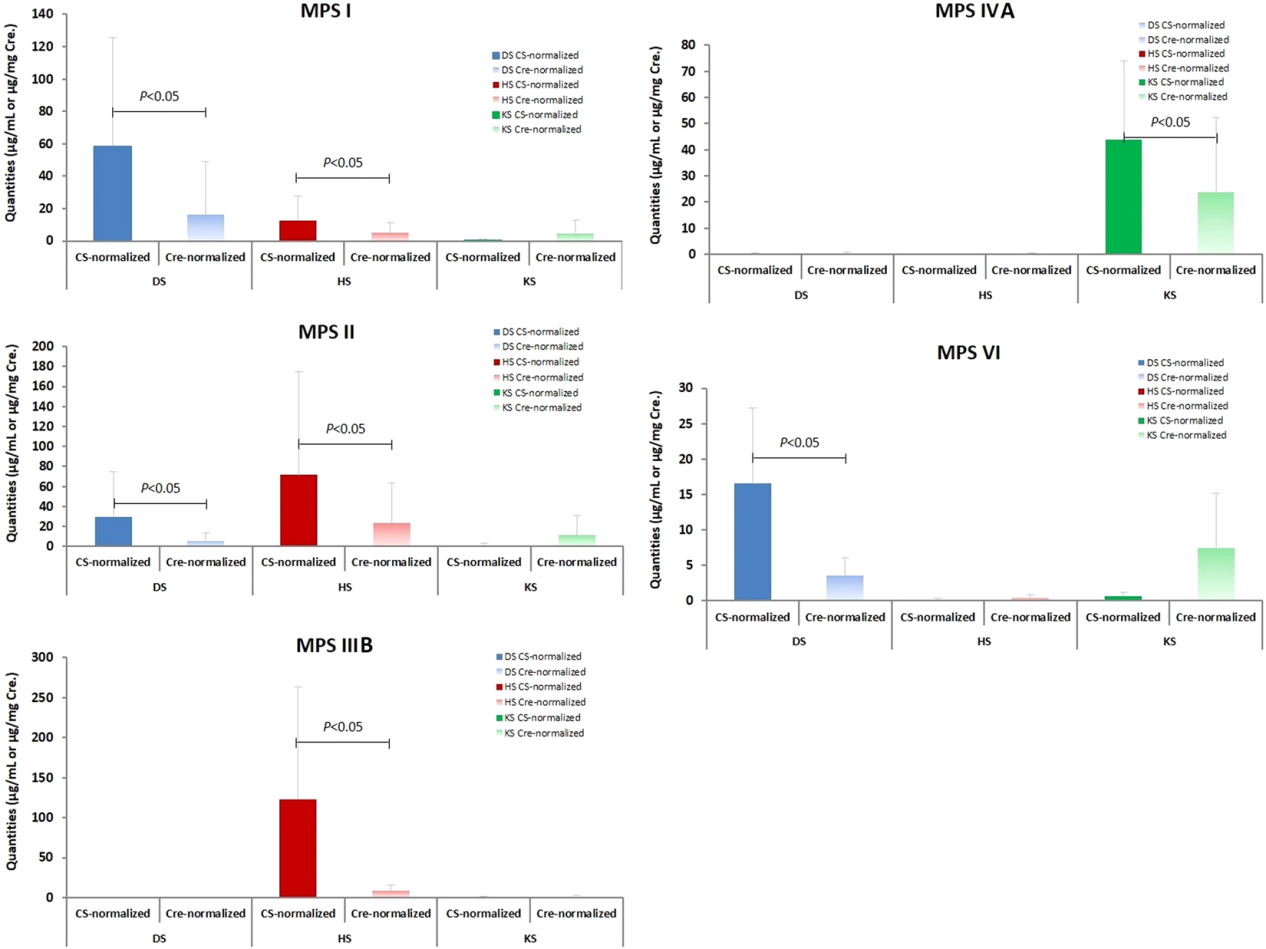
Comparison showing CS-normalized *vs*. creatinine-normalized methods to determine the quantities of DS, HS, and KS in the patients with different types of MPS. By using the CS-normalized method, the quantities of affected GAGs found in the patients with different types of MPS were more consistent and more noticeable than those calculated using the creatinine-normalized method. Both DS and HS were elevated in the patients with MPS I and MPS II; only HS, KS, or DS were increased in the patients with MPS IIIB, IVA, and VI, respectively.

The type of GAG-derived disaccharide differs according to the type of MPS, with DS and HS found in MPS I and II, HS only in MPS type III, KS only in MPS type IV, and DS only in MPS type VI. The results of CS-normalization to quantitatively measure the levels of GAG-derived disaccharides consistently showed these associations, however using creatinine-normalization showed inconsistencies in some of these associations along with variations in urine creatinine level (Fig. 2). Furthermore, false negative results were frequently observed using the creatinine-normalized method, which could result in an incorrect MPS diagnosis or outcome survey after ERT.

### Results of MPS I

Thirteen confirmed patients with MPS I aged from 0.7 to 38.2 years were analyzed, including four cases with MPS I identified during newborn screening in Taiwan without clinical presentations, six cases with the Scheie phenotype, and another three with Hurler-Scheie disease. The main affected GAGs in these patients were DS and HS. In this study, the DS and HS values using CS-normalization ranged widely from 4.10 to 209.12 μg/mL, and from 0.15 to 40.91 μg/mL, respectively. The results were in good agreement with the actual severity in the patients with MPS I and with pre- or post-ERT. In comparison, creatinine-normalization showed positive levels of DS and/or HS in most cases, but not as significantly as with CS-normalization due to the influence of large variations in urine creatinine levels. Statistical analysis (Students T-test) of the two normalized methods showed *p* values of 0.027 and 0.049 for DS and HS, respectively, and the differences were statistically significant (<0.05). Of note, few cases (3/13; patient # 9, 10, and 11) showed false negative results due to the higher influence of urine creatinine (Supplementary Table 1).

### Results of MPS II

A total of 25 confirmed patients with MPS II were analyzed, including six infants with suspected MPS II without clinical manifestations, nine with the mild form, four with the intermediate form, and six with the severe form. Their ages ranged from 0.2 to 28.9 years. The values of DS, HS, and even KS calculated by either CS-normalized or creatinine-normalized methods varied widely (from 1.60 to 170.76 μg/mL for DS; from 1.94 to 384.03 μg/mL for HS using CS-normalized method) due to the severity of the disease and whether or not the patients had received ERT. In general, lower DS values and higher HS values were noted in comparisons between patients with MPS I and II. The DS and HS values obtained from the CS-normalized method were in excellent agreement with the actual severity of the MPS II patients. Using creatinine-normalization, some DS and HS values may have been underestimated in some cases (6/25) which may have resulted in false negative interpretations (Supplementary Table 2). Moreover, a few cases (7/25) showed false positive results in KS quantification. Statistical analysis (Students T-test) of the two normalized methods showed *p* values of 0.010, 0.017, and 0.011 for DS, HS, and KS, respectively, and the differences were statistically significant (<0.05).

### Results of MPS IIIB

Eleven patients with confirmed MPS IIIB with significantly affected central nervous systems were analyzed, with ages ranging from 4.3 to 22.7 years. An increase only in HS level was characteristic of these patients. The values of HS calculated using the CS-normalized method were significantly elevated, with a wide range from 8.75 to 500.43 μg/mL, which may have been due to the phenotype of MPS IIIB. A few cases (4/11) showed false negative results due to high concentrations of urine creatinine being detected (92.63 ~ 312.32 mg/dL) (Supplementary Table 3). Statistical analysis (Students T-test) of the two normalized methods for HS showed a *p* value of 0.027, and the difference was statistically significant (<0.05).

### Results of MPS IVA

Twelve patients with confirmed MPS IVA were enrolled in this study, all of whom had devastating manifestations including systemic skeletal dysplasia, short stature, joint abnormalities, malformation of the thorax, and valvular heart disease. The ages of the patients ranged from 0.7 to 32.1 years, and the KS levels were remarkably elevated (from 11.04 to 103.65 μg/mL) when calculated using CS-normalization compared with the cut-off value (<7.81 μg/mL); whereas, the increases in KS levels were inconsistent calculated using creatinine-normalization due to large variations in urine creatinine levels (from 27.54 to 231.65 mg/dL). The higher the urine creatinine levels, the lower the calculated quantity of KS. Five of the 12 patients showed false negative results (<13.13 μg/mg creatinine) which would have led to significant misinterpretation of the data for an MPS IVA patient or follow-up outcome survey after ERT (Supplementary Table 4). Statistical analysis (Students T-test) of the two normalized methods for KS showed a *p* value of 0.017, which was statistically significant (<0.05).

### Results of MPS VI

Six patients with confirmed MPS VI (age ranging from 12.5 to 28.4 years) with soft tissue involvement and skeletal abnormalities were enrolled. The only affected GAG in these patients was DS. The DS values were significantly elevated when calculated using CS-normalization compared with the cut-off value (<0.49 μg/mL); whereas the DS values calculated using creatinine-normalization were correspondingly lower. This may have affected the interpretation of the results with regards to diagnosing MPS VI and outcomes after ERT. In addition, one of the six patients had false negative results in DS quantification, and two patients had false positive results in KS quantifications (patient No. 2 and No. 3) (Supplementary Table 5). Statistical analysis (Students T-test) of the two normalized methods for DS showed a *p* value of 0.049, and the difference was statistically significant (<0.05).

### Percentage of GAGs in the urine of the MPS patients and non-MPS controls

**Figure 3:**
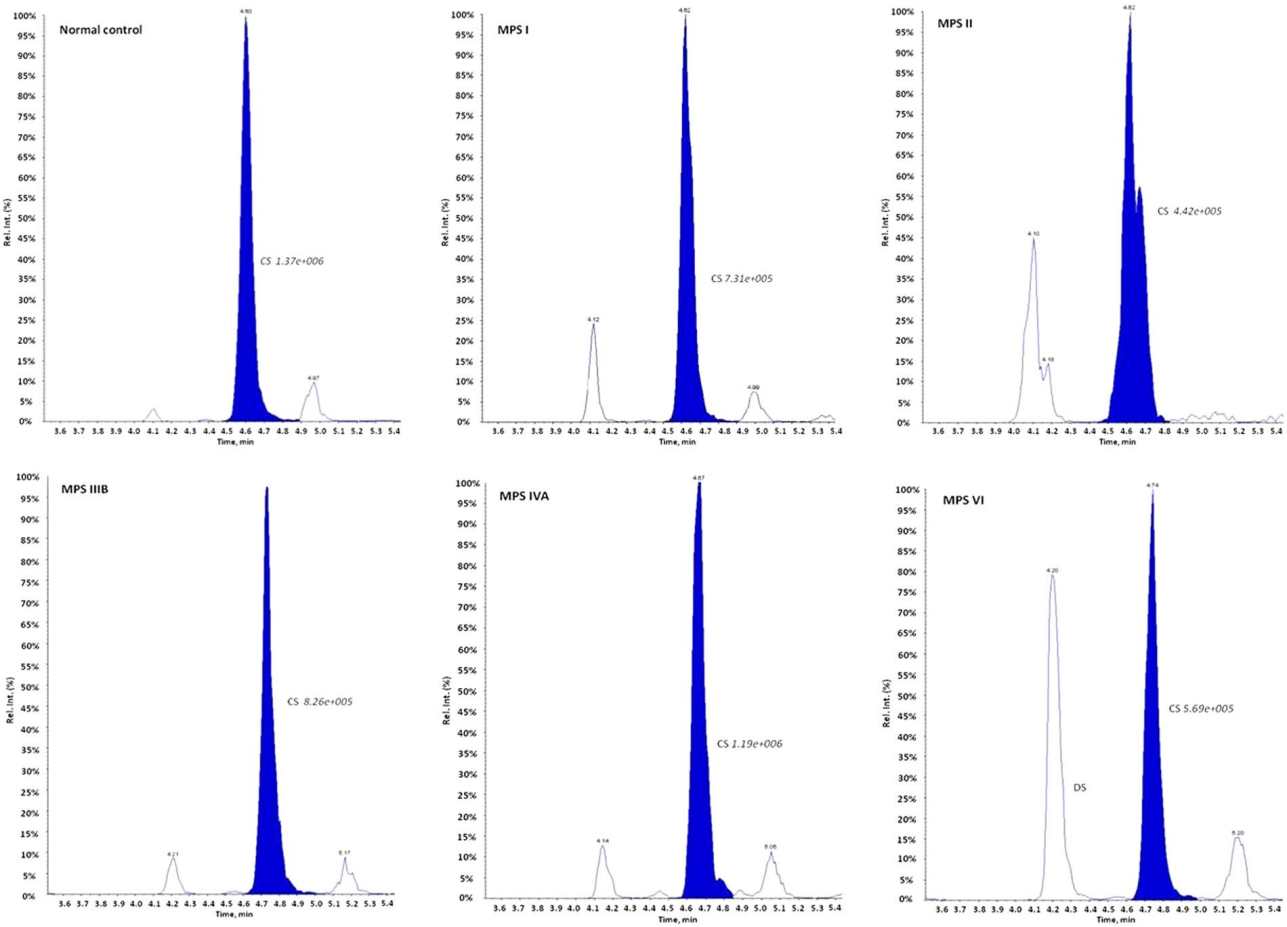
The integrated peak areas of CS obtained from non-MPS controls and from the patients with different types of MPS were similar and this demonstrated that normalizing the GAG-derived disaccharides (HS, DS, and KS) to the excretion of CS is better to discriminate between MPS patients and control individuals, regardless of the creatinine value in the urine.

**Figure 4:**
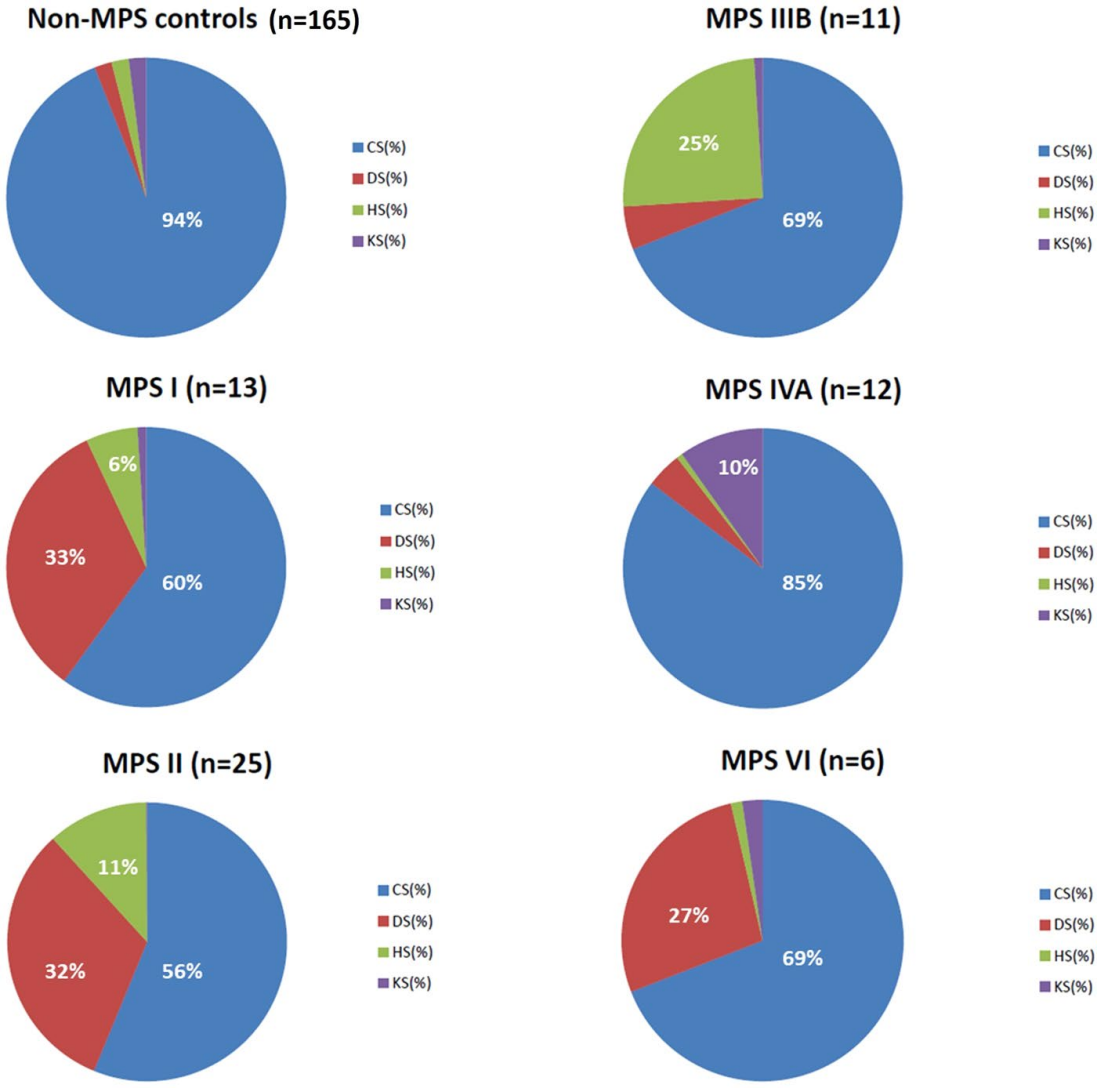
Chondroitin sulfate (CS) was the main constituent of total GAGs in the urine of non-MPS controls, accounting for 94% (±3%). The percentages (%) of CS in the patients with MPS were reduced due to the increased percentages of DS, HS, or KS according to the type of MPS; however, the quantities of CS did not change significantly.

CS was the main GAG constituent (94.0% ± 3%) in the urine of the non-MPS controls, with DS, HS, and KS each accounting for less than 2%. In addition, the quantities of CS found in the non-MPS controls and MPS patients were very similar, as shown by the CS peak areas (Fig. 3). The average CS peak areas found in the non-MPS controls (1.37e + 006) and patients with different types of MPS were similar (ranging from 4.42e + 005~1.19e + 006 on average), accounting for 60.0% (±20%), 56.0% (±21%), 69.3% (±13%), 85.3% (±7%), and 69.1% (±4%) in the patients with MPS I, II, IIIB, IVA, and VI, respectively (Fig. 4).

## Discussion

In this study, CS was the major GAG constituent in the urine of the non-MPS controls, with DS, HS, and KS each accounting for less than 2%. In addition, the volume of CS was found to be inconsistent. Importantly, the reduced percentage of CS found in the MPS patients (Fig. 4) did not mean that the quantity of CS was also diminished. That is, the quantities of CS found in the non-MPS controls and MPS patients were very similar, as shown by the CS peak areas (Fig. 3). These decreases in the percentages of CS were accompanied by increases in the other urinary GAGs (DS, HS, or KS). The total amounts of GAGs were dramatically increased in the patients with MPS I, II, and VI, and moderately elevated in the patients with MPS IIIB and IVA compared to the other types of MPS and non-MPS controls. The differences in the reductions of CS (%) in the patients with different types of MPS were mainly due to increases in the other GAGs associated with that type of MPS. For example, significant elevations in both DS and HS (%) were found in the patients with MPS I and II of about 39.0% and 43.5%, respectively, compared to the normal controls (<4%). Similar results were found for MPS IIIB, IVA, and VI (Fig. 4). For the patients with MPS IVA, the percentage of CS was 85.3%, which was significantly higher than those of the other MPS types, and was mostly due to the defect in step-wise degradation of chondroitin-6-sulfate (C-6-S)^25^. In this study, we did not measure C-6-S or C-4-S separately.

MPS disorders are very heterogeneous, and the accumulations of various GAG-derived disaccharides affect somatic systems, tissues, and organs differently. DS may primarily lead to soft tissue storage and skeletal diseases^26,27^, HS may involve dysfunctions of the central nervous system^28,29^, and KS may principally result in skeletal disorders^30,31^. Notably, KS also is found in non-bone soft tissues^32^. Accordingly, the amounts of GAG-derived disaccharides in different types of MPS correspond well to the severity, clinical manifestations, and onset of MPS disease^23,33,34^. Lin *et al*. reported positive associations between levels of urinary GAG fractionation biomarkers and the phenotypes of MPS, and that the accumulation of one or more specific GAG-derived disaccharides is associated with specific MPS features^35^. For example, patients with intellectual disabilities have significantly higher levels of HS than those without intellectual disabilities, and the DS levels in patients with hernia, hepatosplenomegaly, claw hands, coarse face, valvular heart disease, and joint stiffness are higher than in those without. The MS/MS-based method has been shown to be a more sensitive, specific and reliable tool than dye-based methods to screen and diagnose and identify the type of MPS, and to monitor the efficacy of ERT^35^.

However, it is important to identify the actual amounts of urinary GAG-derived disaccharides as measured by the MS/MS-based method. The conventional unit used to express DS, HS, and KS is μg/mg creatinine according to the creatinine-normalized method. However, when expressed using this unit, the levels of GAG-derived disaccharides vary along with the levels of urine creatinine. In the current study, the false positive rates for both DS and HS in the non-MPS controls were 3.03% (5/165), and the concentrations of urine creatinine were significantly lower in these five controls, ranging from 3.31 to 12.10 mg/dL (mean ± SD = 7.99 ± 3.46 mg/dL). In addition, the false positive rate for KS in the non-MPS controls was 27.3% (45/165). The influence of urine creatinine level was even greater in these patients, leading to significant fluctuations in the quantities of KS, most of which were far greater than the cut-off value (13.79 μg/mg creatinine). However, no false positives were noted for DS, HS, or KS by using the CS-normalized method. An important finding in this study is the false negative results, which were frequently encountered when using the creatinine-normalized method, including 23.1% (3/13) for DS + HS and 30.8% (4/13) for HS in MPS I; 24% (6/25) for DS + HS and 16% (4/25) for DS in MPS II; 36.4% (4/11) for HS in MPS IIIB; 41.7% (5/12) for KS in MPS IVA; and 16.7% (1/6) for DS in MPS VI. In comparison, only a 23.1% (3/13) false negative rate for HS in MPS I patients was found when the CS-normalized method was applied (Supplemental Tables 1–5). Furthermore, many false positives for KS quantification by using creatinine-normalized method were found, particularly in patients with MPS I (2/13), MPS II (5/25), and MPS VI (2/6).

**Figure 5:**
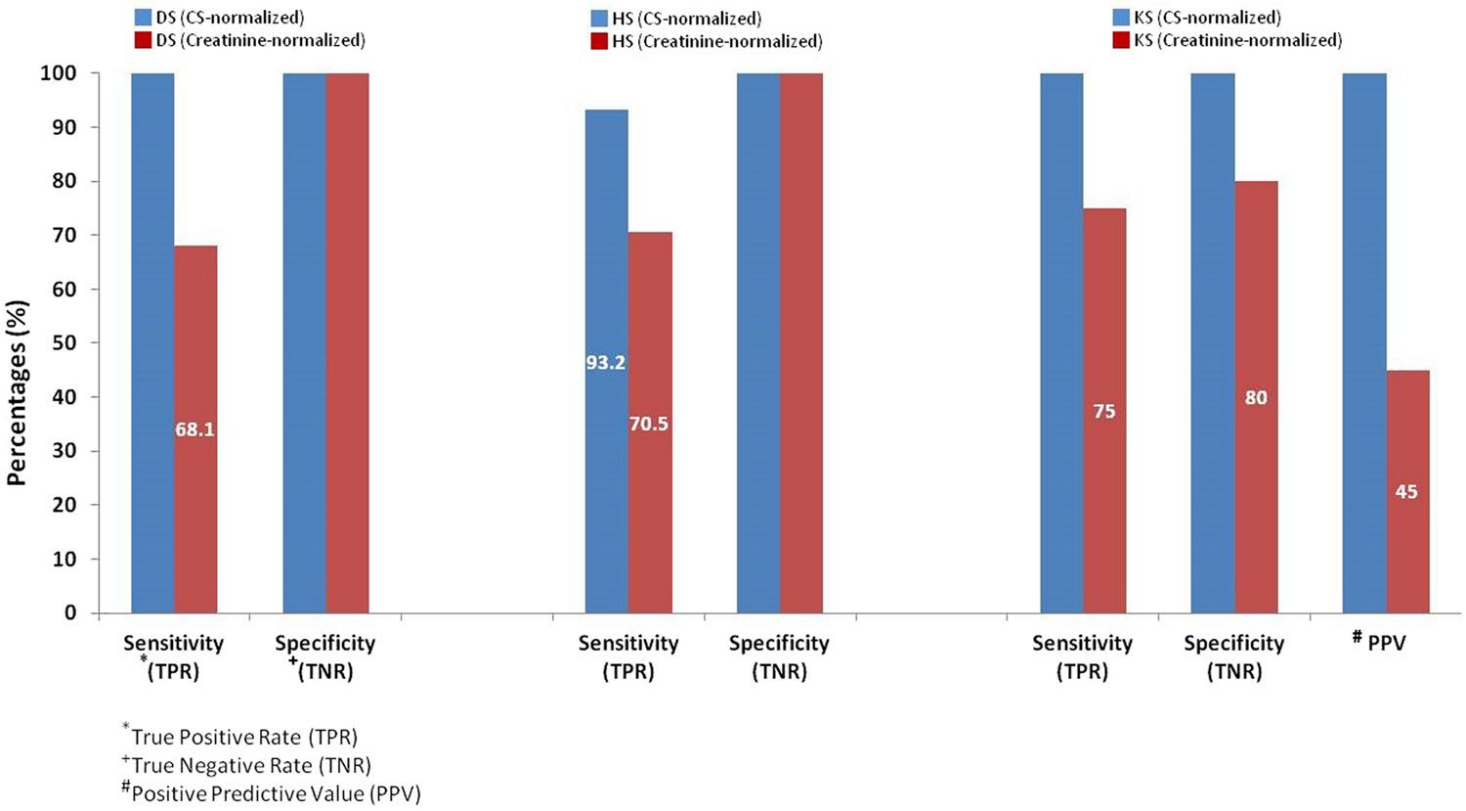
Cross-reactivity on sensitivity (true positive rate; TPR) and specificity (true negative rate; TNR) of the two normalization methods demonstrated that higher false negative (lower TPR and high TNR) rates were found using the creatinine-normalized method when calculating the quantities of DS, HS, and KS.

Cross-reactivity impacted the sensitivity (true positive rate) *vs*. specificity (true negative rate) of the two normalized methods. Low sensitivity and high specificity of DS, HS, and KS were found using the creatinine-normalized method (Fig. 5), including a sensitivity of 68.1% for DS and 70.5% for HS. In comparison, the sensitivities for DS and HS were 100% and 93.2%, respectively, using the CS-normalized method. The specificities of both normalized methods were all 100%. These results indicated that the vast majority of MPS cases could be detected using the CS-normalized method but not the creatinine-normalized method. In turn, this could lead to misinterpretation and even misdiagnosis or misclassification of MPS I, II, IIIB or VI, particularly when evaluating the efficacy of ERT. Moreover, the sensitivity, specificity, and positive predictive value for KS using the creatinine-normalized method were 75.0%, 80.0%, and 45.0%, respectively (Fig. 5). Therefore, the creatinine-normalized method would cause a relatively high possibility of false negatives of MPS IVA compared to the CS-normalization method, and would make evaluating the efficacy of ERT more difficult. In this series, there were no variations in urinary GAG measurements using the CS-normalized method in the patients under treatment. In addition, the accuracy rates of the CS-normalized method were 100% for DS, 95.5% for HS, and 100% for KS compared to 79.1% for DS, 80.6% for HS, and 79.1% for KS when using the creatinine-normalized method.

In conclusion, we showed that significant variations in the quantities of GAG-derived disaccharides can be caused by large variations in urine creatinine levels. Urine creatinine itself is a variable factor which is directly proportional to age but inversely proportional to levels of GAG-derived disaccharides. Normalization of the unit used to express the actual quantity of GAG-derived disaccharides is important, and our results showed that normalizing the quantity of GAG-derived disaccharides (HS, DS, and KS) to CS could better discriminate between MPS patients and controls, regardless of the urinary creatinine value. This method was also more accurate to make a diagnosis with no false negatives or false positives, and for evaluating ERT when comparing post- to pre-treatment (the baseline).

## Methods

### Ethical approval

All experimental protocols were approved by the Institutional Review Board of Mackay Memorial Hospital (14MMHIS281 and 16MMHIS152), and the methods were carried out in accordance with the relevant guidelines and regulations. Informed consent was obtained from all participants and/or their legal guardian/s.

### Urine sample preparation

A total of 232 urine samples were collected from 67 patients with confirmed MPS and 165 controls without MPS. Of the 67 patients, 13 had MPS I, 25 had MPS II, 11 had MPS IIIB, 12 had MPS IVA, and six had MPS VI, according to their clinical manifestations and symptoms, leukocyte enzyme activities, and verified pathogenic variations in MPS gene alleles. The non-MPS controls (n = 165) were referred from the out-patient department of Mackay Memorial Hospital and two newborn screening centers in Taiwan for MPS high-risk screening and confirmation. The urine samples (15–30 mL) were randomly collected in sterile plastic urine containers (polyethylene; Nalge Nunc International, USA) with screw caps and stored in a refrigerator at 4 °C before urinary biochemistry analysis. Urine creatinine was measured using an enzymatic method (Beckman DXC-880i) at the Department of Clinical Laboratory Medicine, Mackay Memorial Hospital. MPS diseases were ruled out according to negative GAG first-line biochemistry examination results, including GAG quantification (DMB/Cre ratio), 2-D EP, and GAG-derived disaccharides using a LC-MS/MS assay, the methods of which have been reported previously^17,36^.

### Methanolysis and keratanase II digestion pre-treatment

**Figure 6:**
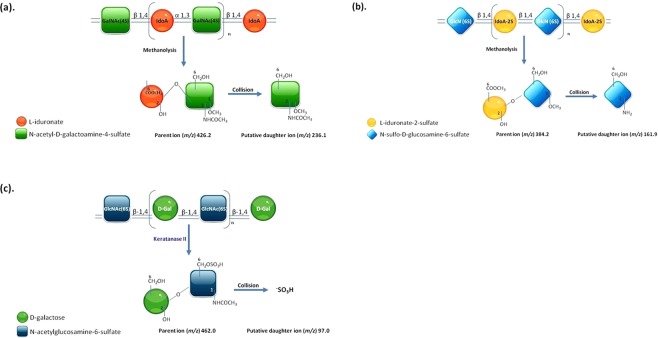
The principles of methanolysis and keratanase II digestion for the quantification of individual GAG-derived disaccharides including DS, HS, and KS by tandem mass spectrometry assay. (**a**,**b**) Present the short hand figures of methanolysis of DS and HS, respectively, and (**c**) presents the principle of keratanase II digestion on KS.

The LC-MS/MS assay for relevant GAG-derived disaccharides was performed using methanolysis for CS, DS, and HS^17–19^ and digestion with keratanase II for KS as described previously^20,21,37^. In brief, the GAGs were precipitated and then degraded to uronic acid-*N*-acetylhexosamine dimers. The *m/z* (mass to charge) of the parent ion and its daughter ion after collision was 426.1 → 236.2 for DS and 384.2 → 161.9 for HS (Fig. 6a,b).

Details of the LC-MS/MS assay for KS-derived disaccharides have been reported by Martell *et al*.^20^ and Oguma *et al*.^21^. In this study, keratanase II was purchased from GlycoSyn (Lower Huff, New Zealand). Keratanase II treatment cleaves *N*-acetylglucosamine linkages of the KS chain, releasing Galβ1-4GlcNAc (N-acetylglucosamine) disaccharides with mono-sulfates. One particular disaccharide of KS was selected. In multiple reaction monitoring (MRM) mode, the mass spectrometer detected ions by monitoring the decay of the *m/z* 462 precursor to the *m/z* 97 production of Gal β1 → 4 GlcNAc(6 S) disaccharides derived from KS (Fig. 6c). The transition *m/z* of the parent ion and its daughter ion after collision was 462.0 → 97.0, whereas that for chondrosine (GlycoSyn; Lower Huff, New Zealand), which was used as an internal standard in this assay, was 353.9 → 73.0^20,21,37^.

### LC-MS/MS assay and calibration

Experimental parameters of LC-MS/MS assay have been reported previously^17,20,21,37^. LC-MS/MS analysis was performed on an AB 4000 QTRAP LC-MS/MS System (AB Sciex, Foster City, CA, USA) equipped with a TurboIonSpray (electrospray ionization; ESI), and Agilent 1260 Infinity HPLC pump and autosampler (Agilent Technologies, Santa Clara, CA, USA). An Atlantis dC18 3 μm column (3.0 × 50 mm; Waters Corporation, Milford, MA, USA) was used for DS and HS analysis, and a Luna 5 μm Silica column (50 × 2.0 mm; Phenomenex Inc., CA, USA) was used for KS analysis. Data were acquired and processed using Analyst 1.5.2^TM^ software (AB Sciex). The intra- and inter-assay precision values of this MS/MS-based method were estimated and determined using three different known concentrations (low, medium, and high levels) of samples in triplicate over a period of 6 days regularly^17^. Calibrations of DS, HS, and KS standards, internal standards, and pooled sample controls with known concentrations were performed with every batch analysis. The experimental parameters of mass spectrometer were checked routinely to ensure the quality of the MS measurement.

### Calculations of GAG-derived disaccharides in μg/mL vs. μg/mg creatinine

Two different methods were used to calculate the levels of DS, HS, and KS. The first used urinary CS as the basis to determine the quantities of urinary DS and HS in μg/mL. In the general population, CS is the main component of GAGs in urine compared to DS, HS and KS. Therefore, using CS as the basis for calculations allows for the objective determination of the actual status of DS and HS while avoiding negative factors such as age, urine creatinine value, and other physiological conditions. The levels of the other GAG-derived disaccharides (i.e. DS, HS, and KS) were calculated according to the calibration curves yielded by the LC-MS/MS assay (analyte peak area/IS peak area *vs*. analyte concentration/IS concentration) multiplied by the individual analyte (i.e. DS, HS, and KS) peak area, and then divided by the CS analyte peak area.

The second method of calculating urinary levels of DS, HS, and KS was in μg/mg creatinine. The quantities of these GAG-derived disaccharides were calculated according to the calibration curves yielded by the LC-MS/MS assay (analyte peak area/IS peak area *vs*. analyte concentration/IS concentration), which were then divided by the level of urine creatinine in mg/dL and multiplied by a dilution factor.

### Statistical analysis

Statistical analysis (Students T-test) was performed to compare values of urinary DS, HS, and KS in individual types of MPS between the CS-normalized and creatinine-normalized methods. The two-tailed *p* values were calculated using Excel 2010 (Microsoft Office), and a *p* value < 0.05 was considered to be statistically significant.

## Supplementary information

Quantitative analysis of GAG-derived disaccharides by LC-MS/MS assay in confirmed MPS patients
