# Supplementary material for: Normalization of glycosaminoglycan-derived disaccharides detected by tandem mass spectrometry assay for the diagnosis of mucopolysaccharidosis
Source: Sci Rep. 2019 Jul 24;9:10755. doi: 10.1038/s41598-019-46829-x (PMC6656773; doi:10.1038/s41598-019-46829-x)
Supplement: Supplementary file 1 — Quantitative analysis of GAG-derived disaccharides by LC-MS/MS assay in confirmed MPS patients [file 41598_2019_46829_MOESM1_ESM.pdf]

Title of the manuscript:

Normalization of glycosaminoglycan-derived disaccharides detected by tandem mass spectrometry assay for the diagnosis of mucopolysaccharidosis.

Author list:

Hsiang-Yu Lin, Yun-Ting Lo, Tuan-Jen Wang, Sung-Fa Huang, Ru-Yi Tu, Tzu-Lin Chen, Shuan-Pei Lin , and Chih-Kuang Chuang.

Supplemental Table 1. Quantitative analysis of GAG-derived disaccharides by LC-MS/MS assay in confirmed MPS I patients

MPS I Patient No.	Age (years)	Severity	Receiving ERT (Years)	CS-normalized			Creatinine† (mg/dL)	Creatinine-normalized		
				DS* (<0.49 μg/mL)	HS* (<0.56 μg/mL)	KS* (<7.81 μg/mL)		DS† (<1.56 μg/mg cre.)	HS† (<3.1 μg/mg cre.)	KS† (<13.13 μg/mg cre.)
**1**	5.2	I (H/S)	4.1	22.13	2.51	1.78	24.36	11.93	6.69	25.66
**2**	15.3	I (H/S)	4.6	5.45	0.35	0.23	75.58	1.89	1.32	6.42
**3**	17.5	I (S)	No ERT	75.72	0.15	0.00	126.55	2.99	0.25	0.00
**4**	2.0	NBS#	No ERT	4.35	9.19	0.00	53.45	2.01	6.59	0.00
**5**	0.7	NBS#	No ERT	4.10	3.38	0.17	21.75	5.24	9.26	16.71
**6**	1.4	NBS#	No ERT	118.46	33.67	0.53	14.76	40.13	11.41	0.18
**7**	1.4	NBS#	No ERT	159.87	28.91	0.33	6.66	120.02	21.70	0.25
**8**	26.6	I (S)	4.4	18.98	2.14	1.70	47.37	2.00	0.23	0.18
**9**	38.6	I (S)	3.7	8.48	0.63	0.25	325.61	0.13	0.01	0.00
**10**	37.2	I (S)	4.7	5.19	0.45	0.04	133.18	0.19	1.12	0.00
**11**	21.5	I (S)	6.5	13.34	25.09	0.06	127.59	1.03	1.10	1.57
**12**	2.6	I (H/S)	0.4	68.84	31.26	0.31	98.32	3.43	3.62	4.47
**13**	16.7	I (S)	No ERT	209.12	40.91	0.39	134.92	2.79	1.22	1.89

*using CS as a basis for GAG-derived disaccharide calculation;

†using mg creatinine as a basis for GAG-derived disaccharide calculation;

#confirmed MPS I infants from Newborn Screening Centers in Taiwan;

The reference cut-off values were determined in a range of +3SD

The *p* values (Students T-test) of DS and HS were 0.027 and 0.049, respectively ($p<0.05$) when comparing the CS-normalized method to the creatinine-normalized method.

Supplementary Table 2. Quantitative analysis of GAG-derived disaccharides by LC-MS/MS assay in confirmed MPS II patients

MPS II Patient No.	Ages (years)	Severity	Receiving ERT (Years)	CS-normalized			Creatinine† (mg/dL)	Creatinine-normalized		
				DS* (<0.49 μg/mL)	HS* (<0.56 μg/mL)	KS* (<7.81 μg/mL)		DS† (<1.56 μg/mg cre.)	HS† (<3.1 μg/mg cre.)	KS† (<13.13 μg/mg cre.)
**1**	13.6	Mild	8.3	3.56	11.44	6.89	79.81	1.07	3.26	11.72
**2**	2.7	Severe	No ERT	15.17	346.04	1.43	45.20	2.29	24.34	9.33
**3**	0.9	NBS[#]	No ERT	21.21	12.06	6.47	15.38	6.90	3.92	2.10
**4**	22.7	Mild	6.8	21.90	2.09	1.64	255.52	0.43	0.04	0.03
**5**	24.1	Mild	10.9	2.95	1.94	5.38	238.01	0.20	0.27	3.13
**6**	4.7	Mild	No ERT	36.66	234.38	2.82	42.64	4.40	22.51	8.42
**7**	0.4	NBS[#]	No ERT	18.92	103.44	1.27	4.36	35.55	177.75	95.07
**8**	29.0	IM	6.9	9.71	23.89	2.07	91.08	1.31	4.22	5.27
**9**	28.0	Mild	10.5	2.58	7.60	0.15	302.58	0.21	0.72	0.74
**10**	15.0	Mild	10.5	3.00	2.15	0.18	122.32	0.66	1.52	3.63
**11**	0.3	NBS[#]	No ERT	21.40	30.93	0.09	10.63	16.09	115.71	36.50
**12**	10.5	Mild	3.7	1.81	2.66	0.22	92.93	0.64	1.30	4.56
**13**	25.3	IM	7.1	170.76	61.39	0.00	19.80	11.87	33.08	1.96
**14**	14.0	IM	9.6	8.35	77.72	0.56	95.64	0.90	7.42	2.32
**15**	2.7	IM	No ERT	41.27	167.46	0.18	14.01	14.06	36.40	26.05
**16**	6.4	Severe	No ERT	159.47	152.00	0.35	42.20	6.20	24.76	4.24
**17**	0.2	NBS[#]	No ERT	11.93	175.36	0.08	7.83	15.01	42.35	29.31
**18**	4.5	Mild	Unknown	3.14	27.44	0.14	46.16	1.97	8.24	13.65

**19**	41.1	Mild	8.1	7.59	28.45	0.28	148.80	0.88	1.48	2.18
**20**	6.5	Severe	2.7	33.85	9.68	0.05	58.86	3.80	8.47	2.99
**21**	0.9	NBS[#]	No ERT	1.60	6.45	0.03	96.12	0.64	5.36	1.46
**22**	3.4	NBS[#]	No ERT	2.87	4.20	0.02	47.16	1.74	9.40	3.54
**23**	10.2	Severe	No ERT	91.93	384.03	0.16	35.72	8.9	17.08	6.47
**24**	3.4	Severe	1.1	13.27	47.98	0.20	26.32	6.91	22.42	18.2
**25**	9.5	Severe	No ERT	65.11	332.58	0.08	17.10	19.33	80.7	15.73

*using CS as a basis for GAG-derived disaccharide calculation;

†using mg creatinine as a basis for GAG-derived disaccharide calculation

[#]confirmed MPS II infants from Newborn Screening Centers in Taiwan;

The reference cut-off values were determined in a range of +3SD.

The *p* values (Students T-test) of DS, HS, and KS were 0.010, 0.017, and 0.011, respectively ($p<0.05$) when comparing the CS-normalized method to the creatinine-normalized method.

Supplemental Table 3. Quantitative analysis of GAG-derived disaccharides by LC-MS/MS assay in confirmed MPS IIIB patients

MPS IIIB Patient No.	Ages (Years)	Severity	Receiving ERT (Years)	CS-normalized			Creatinine† (mg/dL)	Creatinine-normalized		
				DS* (<0.49 μg/mL)	HS* (<0.56 μg/mL)	KS* (<7.81 μg/mL)		DS† (<1.56 μg/mg cre.)	HS† (<3.1 μg/mg cre.)	KS† (<13.13 μg/mg cre.)
**1**	9.5	N/A	No ERT	0.16	22.16	0.01	99.07	0.13	4.87	0.73
**2**	4.3	N/A	0.42 (clinical trial)	0.00	92.42	3.59	93.44	0.00	4.95	0.19
**3**	6.3	N/A	No ERT	0.00	48.59	2.68	92.63	0.00	2.62	0.14
**4**	5.3	N/A	No ERT	0.73	179.54	0.57	75.67	0.05	11.86	0.04
**5**	5.7	N/A	No ERT	0.30	74.30	0.36	25.8	0.06	14.40	0.07
**6**	8.3	N/A	No ERT	0.08	20.88	0.03	49.03	0.18	15.40	3.14
**7**	5.4	N/A	No ERT	0.11	8.75	0.03	94.01	0.13	4.6	1.55
**8**	4.3	N/A	No ERT	0.25	38.43	1.03	312.32	0.09	2.23	3.14
**9**	5.3	N/A	No ERT	0.18	271.56	0.02	26.31	0.47	24.71	3.61
**10**	13.2	N/A	No ERT	0.44	500.43	0.52	288.07	0.23	1.96	0.79
**11**	22.7	N/A	No ERT	0.40	94.16	0.69	100.28	0.27	1.27	1.04

*using CS as a basis for GAG-derived disaccharide calculation;

†using mg creatinine as a basis for GAG-derived disaccharide calculation

The reference cut-off values were determined in a range of +3SD.

The *p* value (Students T-test) of HS was 0.027 ($p<0.05$) when comparing the CS-normalized method to the creatinine-normalized method.

Supplementary Table 4. Quantitative analysis of GAG-derived disaccharides by LC-MS/MS assay in confirmed MPS IVA patients

MPS IVA Patient No.	Ages (Years)	Severity	Receiving ERT(Years)	CS-normalized			Creatinine† (mg/dL)	Creatinine-normalized		
				DS* (<0.49 µg/mL)	HS* (<0.56 µg/mL)	KS* (<7.81 µg/mL)		DS† (<1.56 µg/mg cre.)	HS† (<3.1 µg/mg cre.)	KS† (<13.13 µg/mg cre.)
**1**	16.8	N/A	3.1	0.42	0.03	35.92	94.51	0.02	0.00	1.90
**2**	7.1	N/A	No ERT	0.04	0.09	11.04	163.20	0.00	0.17	10.11
**3**	32.1	N/A	No ERT	0.00	0.08	53.24	231.65	0.00	0.00	1.15
**4**	23.1	N/A	6.3	0.24	0.08	12.09	96.85	0.01	0.00	0.62
**5**	20.0	N/A	6.1	0.49	0.08	60.15	36.55	0.07	0.01	8.23
**6**	28.2	N/A	No ERT	0.24	0.01	102.16	27.54	0.04	0.00	18.55
**7**	1.9	N/A	No ERT	0.04	0.00	103.65	39.70	0.00	0.01	109.57
**8**	8.6	N/A	5.8	0.14	0.01	37.11	151.02	0.00	0.17	25.36
**9**	28.2	N/A	2.6	0.82	0.24	23.37	39.10	2.12	0.68	15.60
**10**	7.3	N/A	2.6	0.57	0.12	42.26	81.89	1.25	0.56	27.00
**11**	0.7	N/A	0.6	0.69	0.09	14.95	87.02	0.45	0.33	39.93
**12**	30.6	N/A	0.6	0.43	0.10	32.24	153.04	0.28	0.19	27.39

*using CS as a basis for GAG-derived disaccharide calculation;

†using mg creatinine as a basis for GAG-derived disaccharide calculation

The reference cut-off values were determined in a range of +3SD.

The p value (Students T-test) of KS was 0.017 ($p<0.05$) when comparing the CS-normalized method to the creatinine-normalized method.

Supplementary Table 5. Quantitative analysis of GAG-derived disaccharides by LC-MS/MS assay in confirmed MPS VI patients

MPS VI Patient No.	Ages (Years)	Severity	Receiving ERT (Years)	CS-normalized			Creatinine† (mg/dL)	Creatinine-normalized		
				DS* (<0.49 μg/mL)	HS* (<0.56 μg/mL)	KS* (<7.81 μg/mL)		DS† (<1.56 μg/mg cre.)	HS† (<3.1 μg/mg cre.)	KS† (<13.13 μg/mg cre.)
**1**	12.5	N/A	6.6	34.43	0.01	1.78	94.18	1.83	0.00	0.09
**2**	25.6	N/A	13.9	4.17	0.01	0.86	24.92	2.57	0.12	13.90
**3**	19.3	N/A	11.8	6.67	0.01	0.12	14.70	7.76	1.00	21.67
**4**	19.3	N/A	11.8	12.16	0.09	0.74	220.43	0.74	0.16	2.70
**5**	28.4	N/A	11.6	16.30	0.40	0.25	36.7	2.56	0.88	3.69
**6**	22.0	N/A	12.7	25.90	0.31	0.12	18.97	5.98	0.15	2.87

*using CS as a basis for GAG-derived disaccharide calculation;

†using mg creatinine as a basis for GAG-derived disaccharide calculation.

The reference cut-off values were determined in a range of +3SD.

The *p* value (Students T-test) of DS was 0.049 ($p<0.05$) when comparing the CS-normalized method to the creatinine-normalized method.
